# Innate Immune Regulation by Toll-Like Receptors in the Brain

**DOI:** 10.5402/2012/701950

**Published:** 2012-10-14

**Authors:** Carina Mallard

**Affiliations:** Institute for Neuroscience and Physiology, Sahlgrenska Academy, University of Gothenburg, Box 432, 40530 Gothenburg, Sweden

## Abstract

The innate immune system plays an important role in cerebral health and disease. In recent years the role of innate immune regulation by toll-like receptors in the brain has been highlighted. In this paper the expression of toll-like receptors and endogenous toll-like receptor ligands in the brain and their role in cerebral ischemia will be discussed. Further, the ability of systemic toll-like receptor ligands to induce cerebral inflammation will be reviewed. Finally, the capacity of toll-like receptors to both increase (sensitization) and decrease (preconditioning/tolerance) the vulnerability of the brain to damage will be disclosed. Studies investigating the role of toll-like receptors in the developing brain will be emphasized.

## 1. Introduction

Toll-like receptors (TLRs) were first discovered as the product of the Drosophila gene, *Toll*. It was shown by Dr Nüsslein-Volhard and colleagues that the product of *Toll* in the mother controls the establishment of the dorsal-ventral pattern of the Drosophila embryo [1]. However, 10 years later it was discovered that *Toll* signaling was also involved in the antifungal defence in fruit flies and thereby a link between *Toll* and immunological functions in Drosophila was established [2]. 

The first identified and most extensively studied TLR in mammals is TLR4, which is the receptor that mediates effects by lipopolysaccharide (LPS), the endotoxin secreted by Gram-negative bacteria. It has long been known that the C3H/HeJ strain of mice is resistant to LPS [3, 4]. It was later found that another mouse strain (C57BL/10ScCr) also was resistant to LPS [5]. Mutations in both of these strains were associated with a defect in the *Lps locus*. In 1998 the *Lps locus* was identified to encode the TLR4 receptor [6] and mice with mutations in the *Tlr4* gene were shown to have defective LPS signaling [7]. Since then 13 TLRs have been identified in mouse and human, each reacting to a specific set of signature molecules on different microbes [8]. However, not all TLRs are expressed in both human and mouse. TLR10 exists in humans but not in mouse and the ligand is currently unknown [9]. TLR11, TLR12, and TLR13 are present in mouse but not in human. TLRs are now among the most well-characterized pattern recognition receptors (PRRs) and have been shown to detect a variety of pathogen-associated molecular patterns (PAMPs) as well as endogenous danger signals, so-called damage-associated molecular patterns (DAMPs) [10].

## 2. Toll-Like Receptor Signaling

TLRs are transmembrane receptors containing an extracellular domain, a trans-membrane domain, and an intracellular domain. Upon stimulation the receptors form homodimers or heterodimers. For example, TLR2 can form heterodimer complexes with TLR1 or TLR6 and specific ligands have been identified that can differentiate functions between these TLR1/2 and TLR2/6 complexes. Some receptors are also dependent on non-TLR comolecules. For example, TLR4 activation by LPS requires the comolecules MD2 and cluster of differentiation (CD) 14 [11]. Also TLR2 use accessory molecules such as CD36 [12] and Dectin-1 [13]. 

TLR1, 2, 4, and 5 are localized to the outer cell surface membrane, while TLR3, TLR7, TLR8, and TLR9 are intracellular receptors positioned on endosomes or lysosomes. However, it has become clear over the last few years that extensive intracellular trafficking of the TLRs occurs inside the cell. The endosomal receptors travel from the endoplasmatic reticulum aided by the “helper” protein Unc-93 homolog B1 [14], to the Golgi apparatus and then become activated by proteolytic cleavage by intracellular proteases such as cathepsins in the endosome [15–18]. The compartmentalization of the receptors is believed to be an important mechanism for the organism to distinguish “self” from “nonself” [19].

Downstream of the TLRs different toll/interleukin-1 receptor (TIR) domain containing adaptor molecules links the recognition of microbes to cellular immune responses. The family of TLR-linked adapter proteins is growing and to date includes myeloid differentiation factor-88 (MyD88), MyD88 adaptor-like protein (MAL/TIRAP), TIR domain containing adaptor protein inducing interferon beta (TRIF), TRIF-related adaptor molecule (TRAM), and sterile *α*- and HEAT/armadillo-motif-containing protein (SARM). MyD88 is a common adapter for all TLRs except TLR3 [20]. The TRIF adaptor protein is the sole adaptor protein for TLR3 [21] but is also coupled to TLR4 signaling via the adaptor TRAM [22]. MAL/TIRAP is a bridging adaptor for TLR2 and TLR4 [23]. The fifth adaptor protein, SARM, has been suggested to negatively regulate TRIF [24]. However, with respect to the mouse brain, SARM has been mainly localized to mitochondria in neurons and has been suggested to contribute to cell death [25], which was recently further confirmed [26].

## 3. Toll-Like Receptor Expression in the Brain

Both mRNA and protein expression of TLRs have been identified in the brain. The gene expression for TLR2 and TLR4 was first characterized in the adult rodent brain [27]. Using *in situ* hybridization, it was shown that the mRNA expression for TLR2 and TLR4, under basal conditions, was mainly found in the leptomeninges, choroid plexus, and circumventricular organs (CVOs). These findings were later extended to show an even more widespread expression of TLR4 transcript in the brain, almost exclusively in nonneuronal cells [28]. However, immunoreactivity for TLR2 and TLR4 has later been identified in neural progenitor cells in the hippocampus in adult rodents [29]. Widespread expression of TLRs has also been detected in human cerebral tissue [30]. In this study, microglia, astrocytes, and oligodendrocytes were cultured from human brain and it was found that primary microglia express mRNA encoding for most TLR family members while astrocytes and oligodendrocytes primarily express TLR2 and TLR3. By examining brain and spinal cord tissue sections from control and multiple sclerosis brains, it was observed that TLR3 and TLR4 expression was enhanced in inflamed central nervous system (CNS) tissues. 

TLRs have been studied to a lesser extent in the immature brain; however, recent data suggest that several of the TLRs play a role during brain development. We were the first to demonstrate TLR4 and CD14 gene transcripts in the newborn rat brain [31]. Using reverse transcription-polymerase chain reaction (RT-PCR) we found that while CD14 expression in the brain was strongly induced by systemic LPS, the gene transcript for TLR4 decreased. Later studies have emphasized a role for TLR3 and TLR8 in embryonic brain development. TLR8 immunopositive cells were identified as neurons and stimulation of TLR8 of cortical neurons *in vitro *resulted in inhibition of neurite outgrowth and induced apoptosis [32]. Treatment of cultured embryonic cortical neurospheres with the TLR3 ligand polyinosinic:polycytidylic acid (poly I:C) reduced cell proliferation in a TLR3-dependent manner [33]. In contrast to TLR3 and TLR8, expression of TLR2 is low before birth and increases during the first 2 weeks of life after birth [33]. We showed that mRNA for TLR1–9 is expressed in the neonatal mouse brain and particularly TLR1 and TLR2 transcripts are regulated by hypoxia-ischemia [34]. Further we found that TLR1 protein is expressed in neurons and that TLR2 protein expression is present in astrocytes in the cerebral white matter and in a specific neuronal population in the paraventricular nucleus (PVN) in neonatal mice. In support, the mRNA for TLR1–9 was recently described during mouse development. In this study TLR7 and TLR9 were suggested to be the most developmentally regulated TLRs and the protein expression of TLR7 and TLR9 was identified in neurons in cortex and hippocampus during mouse brain development [35]. 

Taken together, current evidence strongly suggests the presence of TLR gene regulation in cerebral tissue, both during development and after various provocations to the developing CNS. However, the protein expression of TLRs in the brain described in different studies is variable and there appears to be no consensus of which TLRs are expressed and in which cell types. Our own experience is that many of the commercially available antibodies towards TLRs are nonspecific and need further development. 

## 4. Toll-Like Receptors in Ischemic Brain Damage

 As indicated above most of the TLRs are expressed in the brain and are regulated during embryonic and neonatal life, suggesting that they may play a role in brain development. However, it is also becoming increasingly clear that brain TLRs may be important regulators of cerebral ischemia. Tang and colleagues demonstrated that both TLR2 and TLR4 expression is upregulated in cerebral cortical neurons in response to ischemia/reperfusion injury [36]. Similarly, TLR2 is increased in the brain after both permanent and transient focal ischemia [37, 38]. 

Further evidence to indicate that TLRs may contribute to brain ischemia comes from studies demonstrating neuroprotection in TLR-deficient mice. Both the degree of brain damage and neurological deficits observed following permanent and transient middle cerebral artery occlusion (MCAO) are reduced in mice deficient in TLR2 or TLR4 [36, 38–41]. This was later confirmed also in other models of brain ischemia [42, 43]. However, there is one conflicting study reporting that in a focal cerebral ischemia/reperfusion model TLR2 knockout (KO) mice had higher mortality, decreased neurological function, and increased brain infarct size, while TLR4 KO mice were protected from these detrimental processes following ischemia [44]. Interestingly, recent clinical studies have shown that TLR2, TLR4, TLR7, and TLR8 expression in blood was associated with poor outcome following ischemic stroke [45, 46].

In contrast to TLR2 and TLR4, neither TLR3 nor TLR9 deficiency, appears to provide protection from ischemic insults [42]. Neither is there an apparent neuroprotective effect by disruption of the TLR downstream adaptor proteins MyD88 or TRIF against cerebral ischemia [47]. It is intriguing that despite that there are several studies demonstrating neuroprotection in TLR2 and TLR4-deficient animals following cerebral ischemia, KO of the gene for the downstream adaptor molecule, MyD88, does not provide protection. These results open up for the possibility that these receptors might be able to use different downstream adaptors during TLR signaling in the brain. It has for example been suggested that activation of TLR4 results in phosphatidylinositol-3-kinase-(PI3 K-) MyD88 complex formation, and that PI3 K activity selectively leads to cytokine induction downstream of TLR4 [48].

We have studied the role of TLR1, TLR2, TLR4, TRIF, and MyD88 in hypoxic-ischemic brain injury in neonatal mice. In contrast to the adult, we find no neuroprotection in TLR4 deficient mice [49]. Neither have we observed any protection in TLR1 KO [34], MyD88 KO [49], or TRIF KO (Stridh et al., unpublished) animals. However, we did find that mice that were subjected to hypoxia-ischemia on postnatal day 9 demonstrate increased mRNA expression of TLR2 at 6 h and 24 h after ischemia. Immunohistochemical staining of the brains showed that TLR2 was expressed in astrocytes and in a specific population of neurons in the PVN in the hypothalamus. Furthermore, TLR2 KO mice developed smaller infarcts compared to wild-type mice after hypoxia-ischemia [34], indicating a role for TLR2 in the immature ischemic brain.

## 5. Endogenous Toll-Like Receptor Ligands

The presence of TLRs in the brain and their regulation following cerebral ischemia suggest that TLRs can be activated by endogenous TLR ligands. Tissue damage causing cell death and tissue remodeling has been shown to generate endogenous danger molecules, which can act as endogenous TLR ligands [50]. Such endogenous danger molecules, so called DAMPs, have been noted after brain ischemia and include heat shock proteins (HSPs), hyaluronan, nucleic acids, and high mobility group box  1 (HMGB1) [51, 52]. These will be described in more detail below.

### 5.1. Heat Shock Proteins

HSPs are a family of molecular chaperones, which support the correct folding of proteins. HSPs are normally localized in the cytoplasm but are released from necrotic cells. It has been reported that necrotic, but not apoptotic, cell death leads to the release of chaperones such as HSP70, HSP90, calreticulin, and Gp96 [53]. Once outside the cell, extracellular HSPs can have immune-stimulatory properties and have been shown to act as endogenous ligands for TLRs [54–56] and there appears to exist a TLR4-dependent link between the release of HSP60 from damaged cells in the brain and microglia activation [57].

### 5.2. Hyaluronan

Hyaluronan (or hyaluronic acid) is a major component of the extracellular matrix. It is widely distributed in many tissues in the body. Under physiological conditions, hyaluronan exists as a high molecular weight form; however, hyaluronan is actively broken down into lower molecular weight fragments following tissue damage. It has become clear that the function of hyaluronan depends on in which form it exists and low molecular fragments are often seen in pathological situations. The fragmentation of hyaluronan in disease may be a consequence of dysregulation of hyaluronan degradation enzymes [58] or release of reactive oxygen species during tissue damage [59, 60].

The low molecular weight hyaluronan is known to interact with innate immune receptors. Hyaluronan stimulation of dendritic cells is dependent on TLR4 [61] and chemokine and cytokine production in macrophages is abolished in a MyD88- or TLR2/TLR4-dependent manner [62, 63]. Interestingly, although both hyaluronan and LPS activate TLR4, they act via different coreceptors. It was discovered that a unique complex of TLR4, MD2, and CD44 recognizes hyaluronan in noninfectious inflammation, which is different from the TLR4, MD2, and CD14 complex that recognizes LPS during infection [64]. It was further noted that hyaluronan and LPS induce different sets of gene expression, with hyaluronan generating a pattern of gene induction that mimics the response seen after sterile injury with an increase in molecules such as transforming growth factor (TGF) beta 2 and matrix metalloproteinase (MMP) 13. Such genes were not regulated following LPS, suggesting distinct differences between infectious versus noninfectious stimulation of TLR4. 

The effect of high and low molecular weight hyaluronan in brain damage is unclear. High molecular weight hyaluronan accumulates in astrocytes in demyelinated lesions from individuals with multiple sclerosis and in mice with experimental autoimmune encephalomyelitis [65]. It was also shown that the addition of high molecular weight hyaluronan to oligodendrocyte progenitor cultures reversibly inhibits progenitor cell maturation, whereas degrading hyaluronan in astrocyte-oligodendrocyte progenitor cocultures promotes oligodendrocyte maturation. In contrast, in the human brain following cerebral ischemia the low molecular weight hyaluronan form is found [66] and a recent report shows that TLR2 activation by low molecular weight hyaluronan inhibits neurosphere formation *in vitro* [67]. These reports suggest that high and low molecular weight hyaluronan may have differential effects depending on cell type and developmental stage. 

### 5.3. High Mobility Group Box  1

 HMGB1 is released extracellularly during acute inflammatory responses and when membrane integrity is lost in permeabilized or necrotic cells [68, 69]. Thus HMGB1 release is thought to be an important mechanism whereby necrotic cells can trigger inflammation [70]. Park and colleagues reported that stimulation of neutrophils, monocytes, or macrophages by HMGB1 required both TLR2 and TLR4 resulting in increased nuclear translocation of nuclear factor kappa-light-chain-enhancer of activated B cells (NF-*κ*B) and enhanced expression of proinflammatory cytokines [71]. However, *in vitro* studies using primary cells and cell lines demonstrated that the usage of TLR2 and TLR4 in HMGB1 signaling is complex and context dependent [72]. Neutralizing antibodies against TLR4, but not TLR2, dose-dependently attenuated HMGB1-induced interleukin (IL) 8 release in human blood and tumor necrosis factor alpha (TNF-*α*) release in primary macrophages. In contrast, in human embryonic kidney 293 cells transfected with TLR2 or TLR4, HMGB1 effectively induced IL-8 release only from TLR2 overexpressing cells. 

There is evidence to suggest that HMGB1 also acts as a DAMP in the brain following ischemia. Treatment with neutralizing anti-HMGB1 monoclonal antibody ameliorated brain infarction and reduced deficits in locomotor function following 2-hour occlusion of the middle cerebral artery in rats [73]. Anti-HMGB1 treatment inhibited the increased permeability of the blood-brain barrier, activation of microglia, the expression of TNF-*α*, and inducible nitric oxide synthase (iNOS) and suppressed the activity of MMP9. Ischemia-induced release of HMGB1 in brain has later been confirmed by several laboratories [74–76] and appears to be dependent on TLR4 [77]. Recently, another DAMP, peroxiredoxin, was identified in the ischemic brain [78]. Neutralization of peroxiredoxin with antibodies suppressed inflammatory cytokine expression and infarct volume following MCAO. These effects were more pronounced than those seen with inhibition of HMGB1, but interestingly, there were synergistic effects of peroxiredoxin and HMGB1. 

### 5.4. Nucleic Acids

The intracellular TLRs (TLR3, TLR7, TLR8, and TLR9) are located in endolysosomes where they recognize microbial nucleic acids and initiate innate and adaptive immune responses [79, 80]. The subcellular compartmentalization of TLRs is believed to be an important mechanism to prevent autoimmune reactions and block responses to self-nucleic acids [81]. “Self-” nucleic acids, but not viral nuclei acids, are rapidly degraded before reaching endolysosomes, therefore by locating the receptors in the endosomes recognition of “self” is averted [19, 82]. However, both TLR7 and TLR9 are able to respond to endogenous RNA and DNA if they are expressed on the cell surface [83]. TLR3 recognizes viral double-stranded RNA [84], but endogenous mRNA released from necrotic cells has also been shown to activate TLR3 [85, 86]. Therefore, there are situations where possibly TLR3, TLR7, and TLR9 can be activated by endogenous molecules such as “self” RNA and DNA.

Several DAMPs have been recognized to originate from mitochondria following cellular stress [87]. Mitochondria share many features with microbes due to their bacterial origin, suggesting that release of mitochondrial content may act as a TLR activator. As such, mitochondrial DNA (mtDNA) released from dying cells has been identified as a DAMP [88]. mtDNA contains hypomethylated CpG motifs that are similar to bacterial CpG DNA and hence are potent stimulators of TLR9 [89]. mtDNA was recently shown to cause inflammation and heart failure [90]. Interestingly, cerebral hypoxia-ischemia rapidly increases mitochondrial biogenesis in neonatal rats [91]. In this study brain mtDNA content increased 6–24 hours after hypoxia-ischemia, raising the possibility that mtDNA may also be a stimulator of innate immunity in the brain. 

## 6. Effects of Systemic Toll-Like Receptor Ligands on Brain Inflammation 

As discussed above, TLRs are expressed in the brain and endogenous ligands are released following ischemic brain damage, suggesting that innate immune receptors may have a direct effect on brain damage. However, innate immune responses in the circulation also clearly play important roles in generating brain inflammation [28, 92]. Systemically applied LPS initiates a wide range of inflammatory responses in the brain, including increase in cytokines, chemokines, prostaglandins, and nitric oxide. The exposure paradigm seems to play a role as it was shown that in comparison to measurements taken from a time course after a single injection of LPS, repeated injections produced significantly higher cytokine levels in the brain [93]. 

LPS can also affect the integrity of the blood-brain barrier, which could alter interactions between circulating and central mediators. For example, altered immunostaining for junctional proteins b-catenin, ZO-1, and claudin-5; enlargement of intercellular spaces and redistribution of junctional proteins were found in brain endothelial cells *in vitro* after LPS exposure [94]. In the immature brain, Dr. Saunder's group has described the presence of plasma proteins within the white matter tracts of the brain after repeated intraperitoneal injections of LPS (0.2 mg/kg) given at postnatal day 0, 2, 4, 6, and 8. Interestingly, the permeability of the blood-brain barrier to 14C-sucrose and 14C-inulin was still apparent in adult animals that had received serial LPS injections during development. Such changes were not observed after only a single neonatal LPS injection [95]. In later studies, using marsupials, they showed that anti-inflammatory treatment with minocycline restored blood-brain barrier integrity following prolonged LPS-induced inflammation but did not improve LPS-induced damage to white matter. The authors conclude that long-term changes in blood-brain barrier permeability occur only after a prolonged period of inflammation during development; however, damage to white matter can result from even a short-lasting breakdown of the barrier [96]. 

## 7. Transfer of Signal from Circulating Toll-Like Receptors to the Brain

**Figure 1:**
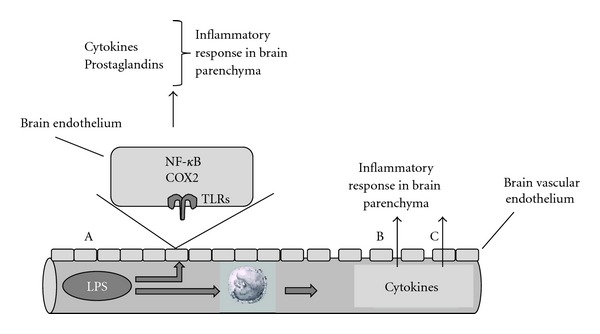
TLR ligand transport across the blood-brain barrier. (A) Circulating LPS binds to endothelial cells in the brain vasculature and transmits inflammatory signals to the brain via COX-2- and NF-*κ*B-associated pathways. Alternatively, LPS in the circulation induces release of cytokines from circulating blood cells, which can either affect the integrity of the blood-brain barrier (B) or be transported across the intact blood-brain barrier (C) to induce further inflammation in the brain parenchyma.

**Figure 2:**
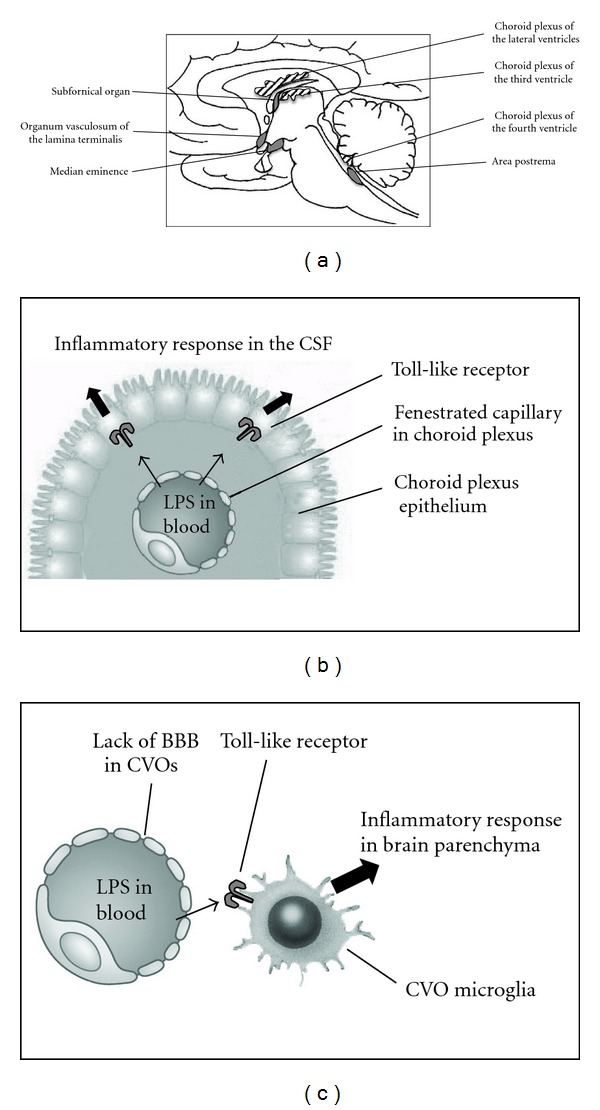
Interaction of systemic TLR ligands with circumventricular organs and choroid plexus. (a) Schematic illustrating the anatomical location of choroid plexus and circumventricular organs (CVO) in the rat brain. (b) Depicted image of suggested transfer of inflammatory stimuli from circulating LPS via the epithelial cells of the choroid plexus and induction of inflammatory responses in the cerebrospinal fluid (CSF) of the brain. (c) The circumventricular organs of the brain lack a fully developed blood-brain barrier (BBB). This allows LPS to instigate interactions with inflammatory cells in these brain regions, which initiates an inflammatory response in brain parenchyma.

There are several potential mechanisms that can explain how TLR ligands induce brain inflammation. In the sections below the following possible scenarios will be discussed: TLR ligand transport across the blood-brain barrier, interaction with brain endothelial cells, interaction with circumventricular organs or epithelial cells in the choroid plexus, and immune transfer via vagal afferents (Figures 1 and 2).

### 7.1. TLR Ligand Transport across the Blood-Brain Barrier

It has long been debated whether circulating LPS, or other TLR ligands, cross the blood-brain barrier to induce brain inflammation. Several recent reports indicate that this is not the case. Iodine-labeled LPS is rapidly cleared (half-life < 30 minutes) from the blood, but there is substantial uptake of LPS by liver, spleen, and lung during this initial period following intravenous injection into rabbits [97]. Tissue-bound LPS was found to be concentrated in phagocytic vacuoles of hepatic Kupffer cells, splenic macrophages, and leukocytes. LPS remaining in plasma beyond 30 minutes was converted to a low-density form, which disappeared from the blood with a half-life of 12 hours. Intraperitoneally injected LPS reaches the circulation within 15 minutes of administration and could potentially also cross the blood-brain barrier [98]. However, after systemic LPS, ions associated with LPS lipids were tracked and found to be bound to brain endothelium but were not found inside the brain, suggesting that LPS does not cross the blood-brain barrier [99]. Similarly, in a carefully conducted study using iodine-labeled LPS, Banks and Robinson recently demonstrated that while intravenously administered LPS binds to endothelial cells of the blood-brain barrier, only minute (<0.025%) levels of LPS were detected inside the brain [100]. This is probably true also for other TLR ligands. A single peritoneal injection of Poly I:C (12 mg/kg) to 8-week-old mice resulted in strong mRNA regulation of a number of chemokines in the brain [101]. Interestingly, blood plasma collected 3 hours after Poly I:C and injected into naive animals also induced an inflammatory response in the brain, suggesting that the presence of Poly I:C itself was not necessary. Further, tracking Poly I:C after injection showed that only minute levels were detected in the blood after intraperitoneal injection. Thus, rather than crossing the blood-brain barrier and directly interact with the brain parenchyma, TLR ligands, such as LPS and Poly I:C, are more likely to induce brain inflammation via indirect mechanisms as outlined below. 

### 7.2. Interaction of Systemic TLR Ligands with Brain Endothelial Cells

The blood-brain barrier, which is formed by the tight junctions of brain capillary endothelial cells, expresses various transporters to regulate exchange of compounds between the brain and the circulating blood [102]. Brain endothelial cells are polarized cells with a luminal (blood-facing) and abluminal (brain-facing) side of the cell membrane, allowing for substances applied to one side to affect release of molecules and regulate transport mechanisms on the other side. Although, TLR ligands, such as LPS and Poly I:C, do not seem to cross the blood-brain barrier, there is ample evidence to suggest that they interact with brain endothelial cells of the barrier and thereby initiate inflammatory responses inside the brain. 

The mRNA for the central immune regulator, I-kappa-B-alpha (I*κ*B*α*), is dramatically increased in the brain after intraperitoneal LPS injection (2.5 mg/kg) [103]. The transcript for I*κ*B*α* was first detected in cells lining the blood side of the blood-brain barrier and then progressed to cells inside brain. The authors suggested that cells of the blood-brain barrier synthesize immune signal molecules to activate cells inside the CNS in response to peripheral LPS. In support, brain endothelial cells isolated from rhesus monkeys that are exposed *in vitro* to either an immune stimulus (IL-1*β* or LPS) or an oxidative challenge (hypoxia) release IL-6 [104]. There is also evidence to show that LPS applied to the abluminal side of brain endothelial cells evokes secretion of IL-6 on the luminal side [105], suggesting a bidirectional release of immune-stimulating mediators across the blood-brain barrier. Furthermore, both IL-1*β* and IL-6 are themselves able to be transported across the blood-brain barrier [106, 107]. Saturable transport systems have also been identified for several other proinflammatory cytokines, including TNF-*α*, leukemia inhibitory factor (LIF), several interleukins, and interferons [108, 109]. However, it is generally believed that the amount of cytokines that are transported across the blood-brain barrier is rather small and play a minor role in affecting brain inflammation in disease. 

Prostaglandin production in cerebral vascular cells has been suggested to be another important interface between peripheral and central inflammation. There is no constitutive expression of cyclo-oxygenase 2 (COX2) mRNA in cerebral blood vessels. However, intravenous injection of LPS induces strong induction of COX2 in blood vessels and the leptomeninges over the entire brain, with the signal maximally enhanced by 50 to 80% over the basal level 1 hour after LPS injection [110]. The route of LPS administration, intraperitoneal or intravenous does not appear to affect the expression [111]. It was later found that it is the interaction between COX2 and microsomal prostaglandin E synthase in brain endothelial cells that is responsible for inducing prostaglandin in the brain [112]. 

Brain endothelial cells express TLR2, TLR4, and CD14 mRNAs [99] and later studies have also shown expression for TLR3 and TLR6 on rat and human cerebral endothelial cells [113]. Convincing data, using chimeric mice, have shown that it is the blood-brain barrier endothelial cells, rather than perivascular microglia, that are the main target of circulating inflammatory mediators to activate the brain response. While the systemic release of acute phase cytokines was dependent on TLR4 on hematopoietic cells, the presence of TLR4 on CNS-resident cells (i.e., nonhematopoietic cells like endothelial cells) was required for sustained inflammation in the brain after systemic LPS administration [28]. Later, these findings were confirmed and extended to show that systemic IL-1*β* caused a robust transcriptional activation of genes involved in prostaglandin E2 production by vascular cells of the brain. Upregulation of these genes was dependent on functional MyD88 signaling in the endothelium, as MyD88-deficient mice that received bone marrow stem cells from wild-type animals (including functional perivascular microglia) exhibited no response to systemic IL-1*β* administration [114].

### 7.3. Interaction of Systemic TLR Ligands with Circumventricular Organs and Choroid Plexus

The CVOs and choroid plexus of the brain have been indicated as important links between systemic inflammation and cerebral innate immune responses. Neurons within the CVOs are activated by intravenous LPS injection [115]. In an early phase following systemic administration of LPS, production of TNF-*α* mRNA was observed in perivascular cells and neurons in CVOs, including the vascular organ of the lamina terminalis, median eminence, and area postrema, as well as along the ventral surface of the medulla in the mouse brain [116]. Later TNF-*α* hybridization was observed over neurons in the hypothalamus and the nucleus of the solitary tract. In support, it has been shown that LPS causes a fast transient rise in intracellular calcium concentrations in the microglial cells in a primary culture of the rat area postrema, with limited responses of neurons, astrocytes, and oligodendrocytes [117]. Similar to TNF-*α*, IL-1*β* production was demonstrated in organum vasculosum laminae terminalis and some cells around the blood vessels in the parenchyma 1 hour after intravenous LPS (4 *μ*g/kg) [118]. Also mRNA expression of IL-6 was detected in the CVOs and choroid plexus following intraperitoneal LPS injection [119]. It was subsequently shown that also TLR4 is present in CVOs and choroid plexus and mediates signals from the periphery by intracellular signaling and then rapid transcription of proinflammatory cytokines, first within these organs and thereafter throughout the brain parenchyma [27]. 

A recent microarray analysis revealed that the mouse choroid plexus displays an acute-phase response after an inflammatory stimulus induced in the periphery by LPS [120]. Genes implicated in immune-mediated cascades and in extracellular matrix remodeling were upregulated, whereas genes that code for protein that participate in maintenance of the barrier function were downregulated. We have evidence to indicate that systemic LPS induces a downregulation of endogenous antioxidant systems in the choroid plexus in neonatal mice (D'Angelo et al., publication under revision). Thus an important mechanism for transducing peripheral inflammation into the brain appears to be by LPS interacting with TLRs in CVOs and choroid plexus. 

### 7.4. Vagal Stimulation by Systemic LPS and Cytokines

Neural afferents, via the vagus nerve, transmit immune messages from the periphery to the brain and contribute to the hyperalgesia, fever, anorexia, taste aversions, increased levels of plasma corticosteroid, and brain norepinephrine changes produced by intraperitoneal injections of IL-1*β*, TNF-*α*, and LPS [121, 122]. These effects seem to be specific to the intraperitoneal route of administration of cytokines because vagotomized animals are still able to respond to IL-1*β* injected intravenously, subcutaneously, and into the lateral ventricle of the brain, but not intraperitoneally [123, 124]. Presently, it is not known whether central cytokine induction via peripheral nerves has any impact on brain damage. 

 In summary, circulating mediators are unlikely to move in sufficient concentrations across barriers of the central nervous system to directly induce major inflammatory processes in the brain. Instead, evidence points towards that peripheral TLR ligands or peripheral cytokines interact with receptors on brain vascular endothelial cells, epithelial cells of the choroid plexus, or cells in the CVOs. The subsequent release of prostaglandin E2 or cytokines into the adjacent brain parenchymal environment appears to be a fundamental step in the relay of blood-borne immune signals to the CNS.

## 8. Role of TLRs in Perinatal Brain Damage

More than 35 years ago, Gilles and Leviton presented the first evidence that LPS endotoxin can cause injury to the developing brain [125, 126]. They showed that a single peritoneal injection of *Escherichia coli* LPS resulted in brain lesions in kittens, neonatal monkeys, and rabbits. The authors further went on and studied infants who died with perinatal telencephalic white matter injury and found that this type of neuropathology was more common in infants who had bacteria isolated from blood at autopsy [127]. These original observations initiated a new field of research and have led to numerous studies over the last few decades into the relationship between inflammation, preterm birth, and perinatal brain damage. Epidemiological and clinical studies have convincingly shown a link between intrauterine infection, neonatal sepsis, and brain damage and the development of cerebral palsy or neurodevelopmental disabilities in children [128–132]. Thus inflammation, either before birth or in the neonate, is now recognized as an important contributing factor in what has been termed “encephalopathy of prematurity” [133].

### 8.1. Maternal Exposure to TLR Ligands

In order to better understand how infection/inflammation can affect the immature brain a number of studies have been performed where the mother, fetus, or newborn animals have been exposed to microbes or bacterial products that act as TLR ligands. Pregnant rabbits that survived inoculation with Escherichia coli developed extensive white matter damage [134, 135]. Animal models of maternal infection have also been developed in rodents. Cai and colleagues demonstrated that cytokines were induced in the rat fetus following maternal LPS administration and neonatal offspring displayed brain damage [136]. Later it was shown that offspring to LPS-treated mothers showed decreased staining for myelin and an increase in astrogliosis [137, 138]. LPS administration to pregnant mice results in extensive gene regulation in the fetal brain, including altered expression of proinflammatory and developmentally regulated genes [139]. A recent study demonstrates that offspring born to LPS-treated dams exhibit reduced social preference and exploration behaviors as juveniles and young adults. In this study, maternal LPS induced dysregulation of genes in the fetal brain belonging to specific functional categories, including increased mRNA expression of cellular stress and cell death genes and reduced expression of developmentally regulated and brain-specific genes, specifically those that regulate neuronal migration of *γ*-aminobutyric acid (GABA)-ergic interneurons [140].

Several studies have been performed to mimic viral infections during pregnancy by giving TLR3 ligands as models for developmentally induced psychiatric disorders [141, 142]. It is now well established that stimulation of the TLR3 receptor during embryonic or fetal development can adversely affect brain development in the offspring. However, the timing of the exposure determines the outcome to some extent [143]. Poly I:C-induced prenatal immune challenge on gestation day 9 but not gestation day 17 significantly impaired sensorimotor gating and reduced prefrontal dopamine D1 receptors in adulthood, whereas prenatal immune activation specifically in late gestation impaired working memory, potentiated the locomotor reaction to the N-Methyl-D-aspartate- (NMDA-) receptor antagonist dizocilpine, and reduced hippocampal NMDA-receptor subunit 1 expression. Pregnant rats given Poly I:C (10 mg/kg), repeatedly during late gestation (E14, E16, and E18), resulted in an increase in monocyte chemotactic protein-1 (MCP-1) in maternal blood 5 hours after injection [144]. In the offspring, the expression of the GluN1 subunits of the NMDA receptors was decreased, but without changes in GluN2A or GluN2B subunits, the postsynaptic density protein 95, or the NMDA receptor modulator EphA4. Also an increase was noted in presynaptic markers such as vesicle-associated membrane protein 1 and synaptobrevin. In contrast, there were no changes in cell proliferation as detected by proliferating cell nuclear antigen or doublecortin. Interestingly, neuropathological consequences of prenatal Poly I:C exposure are exacerbated in offspring with genetic predisposition to dopaminergic abnormalities induced by mutations in the nuclear receptor-related 1 protein [145]. These findings emphasize the importance of gene-environment interactions in these situations. 

### 8.2. Fetal Exposure to TLR Ligands

Direct injection of LPS to fetal sheep results in white matter damage, both in the forebrain [146, 147] and in the cerebellum [148], which is very similar to that observed in preterm infants. Recently, we observed reductions in both white matter volume (~21%) and cortical tissue (~18%) when brains were examined 10 days after LPS exposure in fetal sheep [149]. These neuropathological changes were also confirmed by *ex vivo* magnetic resonance imaging analysis [150]. Importantly, we found that there was loss of the normal maturational increase in cortical electroencephalography amplitude, which correlated with reduced cortical volumes. In the same animal model, we used a global metabolomics approach to examine plasma metabolites differentially regulated by intrauterine inflammation [151]. We detected both acute and delayed effects of LPS on fetal metabolism, with a long-term down-regulation of fetal energy metabolism. The characteristics of the metabolite response to LPS were strongly correlated with white and grey matter volumes at 10 days recovery, suggesting the potential to use metabolomics analysis as biomarker for injury and for identification of therapeutic targets.

### 8.3. Neonatal TLR Exposure

We have studied the effect of inflammation during a developmental stage in the mouse that corresponds to the sensitive period of myelination by injection of either LPS or Pam3CSK4 (TLR1/2 ligand) to mice from postnatal day 3 to postnatal day 11. LPS decreased the serum insulin-like growth factor 1 level on postnatal day 12 and quantification of immunohistochemical staining for axonal, myelin, and oligodendrocyte markers revealed impaired myelination in subcortical white matter. In addition, brain gray matter volume decreased and spleen and liver weight increased at postnatal day 12 [152]. Similarly, mice injected with Pam3CSK4 (5 mg/kg) displayed decreased volume of cerebral gray matter, white matter in the forebrain, and cerebellar molecular layer at PND12 [153]. Such effects were not observed in Pam3CSK4-treated TLR2-deficient mice, indicating a specific TLR2 effect. Systemic Pam3CSK4 injection significantly increased the levels of IL-1*β*, IL-6, chemokine (C-X-C motif) ligand 1, and MCP-1 protein in the brain. The neuropathological changes appear to be transient as there were no long-term effects on memory function, assessed by the trace fear conditioning test at postnatal day 50, nor on the volume of gray or white matter.

## 9. Cerebral Consequences of TLR Ligand Interactions with Other Stimuli

It has long been known that endotoxin can induce a state of tolerance to further infections[154, 155] and numerous studies have shown that different TLR agonists alter inflammatory responses to one another [156, 157]. On the other hand, synergestic induction of TNF production by simultaneous activation of TLR2 and TLR4 has been shown [158, 159] and stimulation of mouse macrophages with both Poly I:C (TLR3 ligand) and CpG DNA (TLR9 ligand) induced more-than-additive levels of TNF, IL-6, and IL-12 p40 [160]. Therefore, it is clear that TLR ligands can induce both priming, synergistic effects on cytokine production as well as tolerance phenomena. The priming or tolerance to cytokine production has been suggested to depend on the TLR adaptor proteins. Thus it was reported that simultaneous and sequential activation of the MyD88- and TRIF-dependent pathways causes synergy and priming, while tolerance is induced by agonists that act through the same pathway [161]. Further, regulation of signaling pathways such as NF-*κ*B [162] and IFN-*γ* [163] has been implicated in the interaction between TLRs.

Compared to systemic effects of TLR cross-reactions, little is known about these processes in perinatal brain damage. Clinical studies indicate that a combination of different etiologies is often present in infants with brain injury [164, 165]. Birth complications are commonly preceded by antenatal infections [166] and the combination of such events dramatically increases the risk of spastic cerebral palsy [132]. However, in a recent study, that performed a systematic review of the literature, it was found that there are both benefits and risks with regard to the effects of chorioamnionitis on brain development of preterm infants [167]. The possibility that TLR stimulation alters the vulnerability of the immature brain [168, 169] and adult brain [170] to injury has been postulated.

### 9.1. TLR-Induced Increase in Vulnerability of the Brain

In order to investigate the interaction between LPS and hypoxic-ischemic brain injury we combined a low sub-sepsis dose of LPS with a subinjury hypoxic-ischemic insult (LPS/HI) in neonatal rats [31]. LPS (0.3 mg/kg) was administered to 7-day-old rats 4 hours prior to 20 minutes of unilateral hypoxia-ischemia. LPS dramatically increased the vulnerability of the immature brain to injury, which could not be explained by a reduction in cerebral blood flow or hyperthermia. In association with the sensitization of injury we found an altered mRNA expression for CD14 and TLR4 in the brain. Subsequently, also direct application of LPS into the brain was shown to increase the vulnerability to hypoxia-ischemia. The combination of intracisternal administration of LPS to 7-day-old rats and hypoxia-ischemia 2 hours later resulted in marked expression of TNF-*α* in the leptomeninges and neuronal injury in the cerebral cortex that was significantly higher than in animals that were subjected to hypoxia-ischemia after intracisternal application of saline [171].

The priming effect of LPS on neonatal brain damage was later shown to be reliant on TLR4 [172] and MyD88 adaptor protein, via microglia stimulation [173, 174]. Several subsequent studies have confirmed the concept of LPS priming on hypoxic-ischemic brain injury in neonatal rodents [175–178]. It remains to investigate how other TLR ligands affect perinatal hypoxic-ischemic brain damage. Our own preliminary observations suggest that stimulation of both TLR2 and TLR3 induces increased vulnerability to subsequent hypoxia-ischemia (Stridh et al., unpublished).

The precise underlying molecular mechanisms of LPS-induced sensitization of brain damage remain unclear. We showed that there is marked gene regulation in the brain following systemic LPS injection in neonatal rats [179]. Gene ontology analysis demonstrated that within the first few hours after LPS, genes associated with protein metabolism, immune and inflammatory responses, chemotaxis, and cell death were overrepresented. We further showed that caspase-3 activity increased and phosphorylation of the Akt kinase decreased in the brain after systemic LPS exposure. Others have shown an imbalance between agonist and antagonist in the IL-1 system, with a shift towards inflammation in LPS/HI brains [180]. The same group has reported that LPS/HI also enhance IL-2 in microglia, but T lymphocytes were not found in the brain [181]. The role of TNF-*α* in LPS/HI-induced brain damage is debated. While the Sebire group has suggested little involvement of this cytokine [182], Kendall and colleagues demonstrated complete prevention of the LPS-induced sensitization of hypoxia-ischemia by deletion of the TNF gene cluster [183]. Interestingly, it was recently shown that LPS preexposure significantly decreased the hypoxia-ischemia-induced tissue-type plasminogen activator (tPA) proteolytic activity but amplified the NF-*κ*B signaling pathway. Anti-tPA therapy lessened microglia activation and brain injury [184]. 

Treatment with corticosteroid improves long-lasting learning impairment following LPS/HI [185]. We later demonstrated that multiple injections of the antioxidant and glutathione precursor N-acetylcysteine (NAC, 200 mg/kg) provided marked neuroprotection, with up to 78% reduction of brain injury, when given both before and after LPS/HI [186]. Protection by NAC was associated with improvement of the redox state and inhibition of apoptosis, suggesting that these events play critical roles in the development of LPS-sensitized hypoxic-ischemic brain injury. We have further investigated the effects of LPS on redox states *in vitro* and showed that conditioned medium from LPS-stimulated microglia induces death of astrocytes, which was associated with down-regulation of the endogenous antioxidant nuclear factor (erythroid-derived 2)-like 2 (Nrf2) system, while there was sustained activation of glycogen synthase kinase 3 beta *β* and p38 mitogen-activated protein kinase [187]. In parallel we noticed decreased acetylation of histone 3 and elevated trimethylation of H3-K9. These effects of microglia-conditioned medium on both the Nrf2 system and the histone acetylation levels were reversed by histone deacetylase inhibitors (HDACs) [188]. Strengthening the possibility that LPS-induced brain damage may cause epigenetic alterations, we recently showed that treatment with the HDAC inhibitor, trichostatin A (TSA), increased acetylation in females after neonatal LPS exposure and reduced grey matter and white matter injury at 5 days post-LPS/HI. Further, TSA treatment altered animal behaviour in the open field and improved learning in the fear-conditioning test in adult females following LPS/HI [189].

### 9.2. TLR-Induced Tolerance in the Brain

In contrast to the tolerance phenomena on cytokine production that develops in the circulation, similar events are not necessarily seen in the brain. In one study, TNF-*α* was repeatedly infused into the lateral ventricle of guinea pig brains. Fever developed after each of the 4 infusions indicating no diminished response to TNF-*α* in the brain [190]. The differential cytokine response in circulation and brain during tolerance has later been confirmed by systemic LPS administration. During endotoxin tolerance, elevation of cytokine expression still occurred in the brain, even when cytokines in the periphery were no longer induced [191]. Similarly, it was recently concluded that innate immune cells in the brain do not become tolerant to systemic infection, but are primed instead, which may lead to prolonged and damaging cytokine production that may have a profound effect on the onset and/or progression of preexisting disease [192].

However, despite the data indicating a lack of tolerance in the brain several studies have demonstrated the development of cross-tolerance between systemic LPS and cerebral ischemia, also called LPS-induced preconditioning. This was first described in adult spontaneously hypertensive rats where LPS was injected prior to permanent MCAO [193]. Infarct volume was significantly reduced by LPS administration 2, 3, or 4 days prior to MCAO. The protective effect of LPS was blocked by coadministration of TNF-binding protein, but not IL-1 receptor antagonist, suggesting that the LPS-induced tolerance to ischemia was mediated by TNF-*α*. It was further shown that the LPS-induced tolerance was not due to attenuation of the ischemic insult by augmenting collateral blood flow, local cerebral blood flow [194]. 

We demonstrated that LPS-induced tolerance to hypoxia-ischemia in neonatal rats was dependent on the exposure paradigm. Thus, rats pretreated with LPS either 6 hours or 72 hours before a short episode of hypoxia-ischemia suffered increased brain damage compared to animals pretreated with saline [195]. In contrast if LPS was administered 24 hours prior to hypoxia-ischemia, brain injury was reduced. Also when hypoxic-ischemic injury was induced 48 hours following LPS, infarct volume was smaller in LPS pretreated animals compared with saline-treated pups [196]. The preconditioning effect of LPS was age related as it was observed in postnatal day 7, 9, and 14 rat pups but not in postnatal day 3 and 5 rats. The effects of neonatal LPS preconditioning are long lasting as long-term followup showed significantly better learning and memory and less brain damage in adult [197].

Many potential mechanisms of LPS-induced tolerance to ischemic brain injury have been suggested. In one study, an increase in superoxide dismutase was observed in association with LPS-induced tolerance in the brain. The beneficial effect of LPS was suppressed by dexamethasone and indomethacin administered 1 hour before LPS, and it was concluded that activation of inflammatory pathways is involved in the development of LPS-induced tolerance [198]. In support of a role for inflammatory mediators in LPS-induced tolerance against ischemia, ceramide, a downstream messenger in TNF-*α* signaling, was shown to be upregulated in the tolerant brain [199]. Also in cultured cerebellar granule neurons, endogenous TNF-*α* seems to be a critical mediator of the neuroprotective actions of LPS independently of the presence of endogenous IL-1*β* [200]. In particular TNF-receptor (TNFR) 1 appears to be important for LPS-induced tolerance as the protective effect of LPS in a model of cell death, induced by oxygen-glucose deprivation in hippocampal slices, was present in tissue from wild-type and TNFR 2-deficient mice, but not in TNFR 1-deficient mice [201]. LPS preconditioning has also been shown to preserve neurovascular function following ischemia [202] and prevent neutrophil infiltration into the brain and microglia/macrophage activation in the ischemic hemisphere [203]. Further, the involvement of adenosine, an endogenous neuroprotectant in the brain after ischemia, has been proposed. In mice that overexpress adenosine kinase, which is the major negative metabolic regulator of adenosine, LPS-induced ischemic preconditioning was abolished [204].

In an interesting series of experiments, Dr. Stenzel-Poore and colleagues have shown the importance of type-1-IFN-related mechanisms in preconditioning of ischemic brain damage in the adult. Microarray analysis of brains collected 24 hours after stroke identified an overrepresentation of type-I-IFN-associated transcriptional regulatory elements in LPS-pretreated animals. These findings were linked to the TRIF pathway as mice that lack TRIF or a TRIF-dependent transcription factor, interferon regulatory factor 3 (IRF3), were not protected by LPS preconditioning [205, 206]. They also investigated the importance of other TLRs in ischemic tolerance and found that systemic administration of the TLR9 ligand CpG oligodeoxynucleotide [207] or TLR3 ligand Poly I:C [208] prior to brain ischemia reduced brain damage. The same group showed that the common denominator in preconditioning by TLR4 and TLR9 ligands as well as brief ischemia, induced genomic changes in the brain characteristic of sequences required for IRF-mediated transcription [209]. 

In neonatal rats, we have found that LPS-induced tolerance was mediated by upregulation of corticosterone in the circulation as RU486, a glucocorticoid receptor blocker, counteracted the LPS-induced tolerance effect and aggravated the hypoxia-ischemia-induced brain injury compared with the vehicle-LPS-treated group [210]. We also found, by gene ontology analysis, that the expression profile in association with tolerance was characterized by over-represented genes belonging to immune and inflammatory processes and cell death/survival genes, including complement component 1, complement component 3, aquaporin 4, epidermal growth factor receptor pathway substrate 15, and PYD and CARD domain containing adaptor protein. Interestingly, there was no indication of a marked type I IFN response in the LPS-preconditioned brain [169]. Hence, this indicates that the molecular cues that mediate preconditioning mechanisms in the immature brain likely differ from those observed in the adult. 

## 10. Effects of Perinatal Inflammation on the Adult Brain

Neonatal LPS alters the neuroendocrine, neurochemical and febrile responses to a subsequent, homotypic (LPS) immune challenge in adults [211–214]. Similarly, animals treated neonatally with Poly I:C have significantly attenuated febrile responses to an adult Poly I:C challenge, which coincided with a heightened corticosteroid response [215]. However, neither neonatal Poly I:C nor neonatal LPS challenges lead to an alteration in the adult febrile or corticosteroid responses to a heterotypic adult immune challenge, indicating that the programming effects of the neonatal immune environment are stimulus specific and do not alter the adult responses to other immune stimuli.

The effect of perinatal immune challenge on adult brain ischemia differs between experimental models. In one study, male Sprague-Dawley rats were subjected to a single injection of LPS at postnatal day 14 and were examined as adults for neuronal cell loss associated with global cerebral ischemia after a two-vessel occlusion. Neonatally LPS-treated rats showed increased cell loss in the central nucleus of the amygdala, a region that is important in the processing of emotional responses. No differences were seen in the CA1, CA3, or dentate gyrus regions of the hippocampus [216]. We subjected mice to intrauterine injection of LPS on gestational day 15. On postnatal day 5, 9, and 70, the offspring were subjected to hypoxia-ischemia. LPS preexposure markedly enhanced brain injury after hypoxia-ischemia in neonatal mice. In contrast, in adult mice, LPS preexposure prevented overall tissue loss after hypoxia-ischemia, but there was still injury to white matter [217]. Neonatal exposure to LPS also impact on experimental autoimmune encephalomyelitis (EAE). Mice exposed to LPS at 2 weeks of age showed a delayed onset and diminished severity of myelin-oligodendrocyte- glycoprotein- (MOG-) induced EAE, induced at 12 weeks. Neuroprotection was associated with an increased number of CD3/forkhead box P3 immunoreactive cells, suggesting early-life microbial exposure influencing the generation of neuroprotective regulatory T cells [218].

Early-life exposure to TLR ligands have also been shown to affect the response to a second immune challenge later in life, which can impact the neural processes underlying memory. In fact, caspase-1 inhibition [219] or inhibiting brain IL-1*β* or microglia activation before the LPS challenge [220] prevents memory impairment in neonatally infected rats. Neonatal LPS exposure also enhances the vulnerability of nigrostriatal dopaminergic neurons to rotenone neurotoxicity in later life suggesting that perinatal brain inflammation may increase adult susceptibility to the development of neurodegenerative disorders [221]. Taken together, there is considerable evidence to indicate that exposure to immune events early in life can impact on a wide range of neurological processes when challenged by similar or different stimuli again in adulthood.

## Abbreviations

BBB: Blood-brain barrier
CD: Cluster of differentiation
CNS: Central nervous system
COX2: Cyclo-oxygenase 2
CVO: Circumventricular organ
DAMP: Danger-associated molecular pattern
EAE: Experimental autoimmune encephalomyelitis
GABA: *γ*-aminobutyric acid
HDAC: Histone deacetylase inhibitor
HSP: Heat shock protein
HMGB1: High mobility group box 1
IFN: Interferon
IL: Interleukin
I*κ*B*α*: I-kappa-B-alpha
iNOS: Inducible nitric oxide synthase
IRF3: Interferon regulatory factor 3
KO: Knockout
LIF: Leukemia inhibitory factor
LPS: Lipopolysaccharide
LPS/HI: LPS and hypoxia-ischemia
MAL/TIRAP: MyD88 adaptor-like protein
MCAO: Middle cerebral artery occlusion
MCP1: Monocyte chemotactic protein-1
MMP: Matrix metalloproteinase
MOG: Myelin oligodendrocyte glycoprotein
mtDNA: Mitochondrial DNA
MyD88: Myeloid differentiation factor-88
NAC: N-acetylcysteine
NF-*κ*B: Nuclear factor kappa-light-chain-enhancer of activated B cells
NMDA: N-Methyl-D-aspartate
Nrf2: Nuclear factor (erythroid-derived 2)-like 2
PAMP: Pathogen-associated molecular pattern
PI3 K: Phosphatidylinositol 3-kinase
Poly I: C: polyinosinic:polycytidylic acid
PRR: Pattern recognition receptor
PVN: Paraventricular nucleus
RT-PCR: Reverse transcription-polymerase chain reaction
SARM: Sterile *α*- and HEAT/armadillo-motif-containing protein
TGF: Transforming growth factor
TIR: Toll/interleukin-1 receptor
TLR: Toll-like receptor
TNF-*α*: Tumor necrosis factor alpha
TNFR: TNF receptor
tPA: Tissue-type plasminogen activator
TRAM: TRIF-related adaptor molecule
TRIF: TIR domain containing adaptor protein inducing IFN beta
TSA: Trichostatin A.
